# Expression and Function of Tetraspanins and Their Interacting Partners in B Cells

**DOI:** 10.3389/fimmu.2018.01606

**Published:** 2018-07-18

**Authors:** Fagui Zou, Xu Wang, Xinxin Han, Gerson Rothschild, Song Guo Zheng, Uttiya Basu, Jianbo Sun

**Affiliations:** ^1^Guanghua School of Stomatology, Hospital of Stomatology, Sun Yat-Sen University, Guangzhou, China; ^2^Guangdong Provincial Key Laboratory of Stomatology, Guangzhou, China; ^3^Department of Microbiology and Immunology, Vagelos College of Physicians and Surgeons, Columbia University, New York, NY, United States; ^4^Department of Medicine, Milton S. Hershey Medical Center at Penn State University, Pennsylvania, PA, United States; ^5^Center for Clinic Immunology, Third Affiliated Hospital at Sun Yat-Sen University, Guangzhou, China

**Keywords:** tetraspanin, B cell, partner, immune regulation, therapy strategy

## Abstract

Tetraspanins are transmembrane proteins that modulate multiple diverse biological processes, including signal transduction, cell–cell communication, immunoregulation, tumorigenesis, cell adhesion, migration, and growth and differentiation. Here, we provide a systematic review of the involvement of tetraspanins and their partners in the regulation and function of B cells, including mechanisms associated with antigen presentation, antibody production, cytokine secretion, co-stimulator expression, and immunosuppression. Finally, we direct our focus to the signaling mechanisms, evolutionary conservation aspects, expression, and potential therapeutic strategies that could be based on tetraspanins and their interacting partners.

## Origin, Development, Features, and Functions of B Cells

### Origin, Subtypes, and Development of B Cells

**Figure 1:**
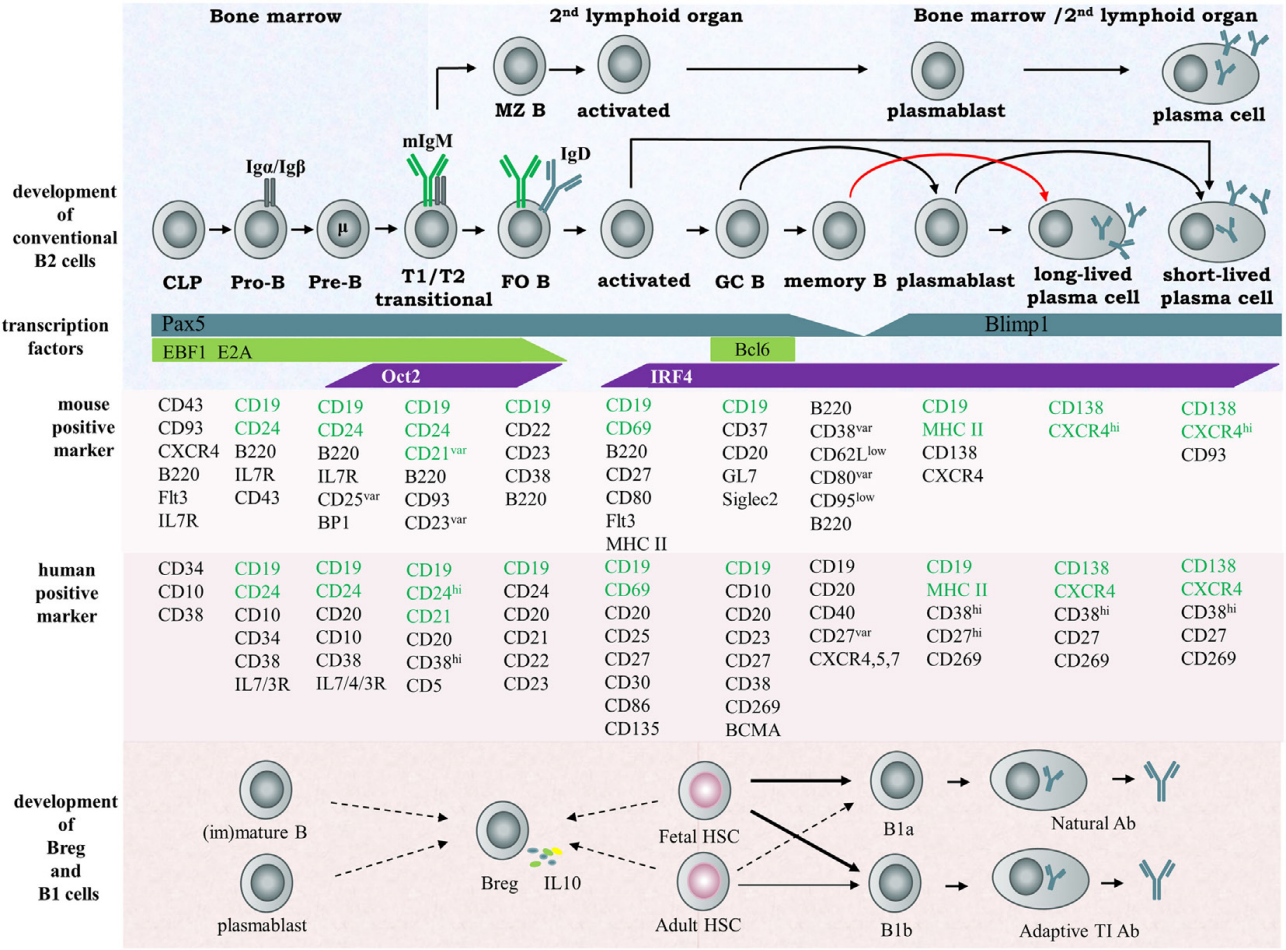
Scheme of B cell development and subsets in humans and mice. According to their individualized origin, surface marker, anatomic localization and functional property, B lymphocytes can be divided into several subsets, including B-1a cell, 1b cell, Breg cell, and B2 cell, the latter considered the conventional B cell. In the early stage, B cells differentiate from hematopoietic precursors into pro-B, pre-B within the bone marrow, then migrate to the spleen and progress through the transitional T1 and T2 stages. These immature cells then differentiate into FO or MZ naïve B cells depending on their special B cell receptor. MZ B cells rapidly develop into plasma cells secreting IgM during the early stage of pathogen infection and function as the first defense line against blood-borne pathogens. FO B cells enter germinal centers and undergo class switch recombination (CSR), somatic hypermuation (SHM), and affinity maturation and terminally differentiate into memory B cells or plasma cells. The important transcription factors and surface markers in human or murine involved in conventional B cell development are shown. The origin of regulatory B cells and B1 cells is still not identified. Here, the solid arrows represent known developmental routes while the dashed arrows represent possible development directions. Abbreviation: CLP, common lymphoid progenitor.

Conventional B cells—a type of white blood cell—were first defined in 1965 by Cooper (1). They originate from hematopoietic stem cells in mammalian bone marrow or in the bursa of Fabricius of birds, where they pass through several developmental stages and become IgM^+^ immature B cells capable of recognizing antigen (1, 2). The immature IgM^+^ B cells subsequently migrate to secondary lymphoid tissues and develop into three groups of mature naïve B cells: follicular B cells, marginal zone B cells (MZB), and B-1b cells. When bound with antigen, mature naïve B cells are activated, selected, and differentiated into plasmablasts and then antibody producing plasma B cells. These are conventional B cells and also named as B-2 cells. There are additional B cell populations (named B-1 cells) generated in the fetal liver or spleen which undergo self-renewal in the periphery and secrete IgM and IgG3 natural antibodies to facilitate immune responses. B-1 cells have a distinct developmental lineage from B-2 cells. The exact origin and development of B-1 cells is uncertain (3). Accumulated evidence indicates the existence of yet other B cells, named regulatory B cells (Breg), and associates their function with suppression of immune responses. Whether Breg is a distinct lineage of B cells is still unknown (4). More details of B cell development stages and B cell subsets are summarized in Figure 1.

### Functions of B Cells

**Figure 2:**
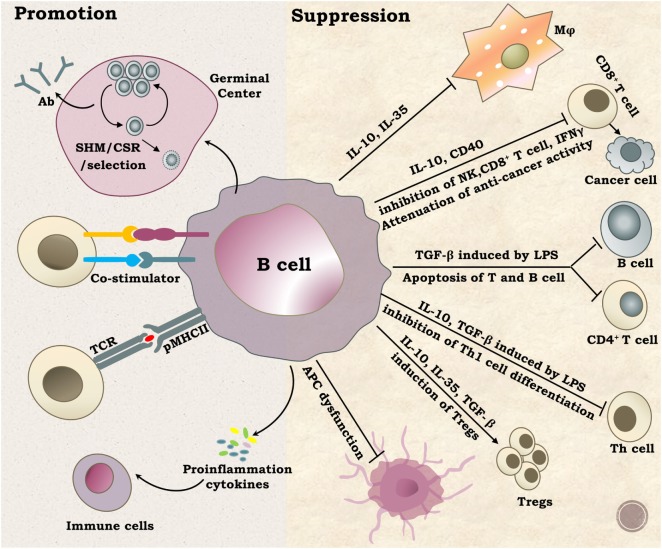
Functions of B cells. B lymphocytes perform diverse and complex roles *in vivo* mainly through promotion or suppression of immune responses. The well-known function of B cells is antibody production by plasma cells after SHM selection and CSR. B cells can also activate other immune cells by providing co-stimulation signals, serving as antigen-presenting cells or secreting multiple proinflammation cytokines, such as IL2, IL4, IL6, TNF-α, and INF-γ. On the other hand, B cells can suppress immune responses by regulating certain types of immune cells through multiple ways. Abbreviations: SHM, somatic hypermutation; CSR, class switch recombination; Ab, antibody.

B cells play pivotal roles in the immune system. As outlined in Figure 2, B cells can promote an immune response through presentation of antigens and production of diverse antibodies, proinflammatory cytokines, and co-stimulators (5). B cells can also suppress immune responses through a variety of mechanisms, such as production of IL-10, IL-35, and TGFb1, induction of regulatory T cells, and clearance of auto antigens (4). Many cell surface molecules are involved in B cell development and function. Tetraspanins are one such important family of molecules.

## General Features and Functions of Tetraspanins

### Structure and Evolutionary Conservation of Tetraspanins

**Figure 3:**
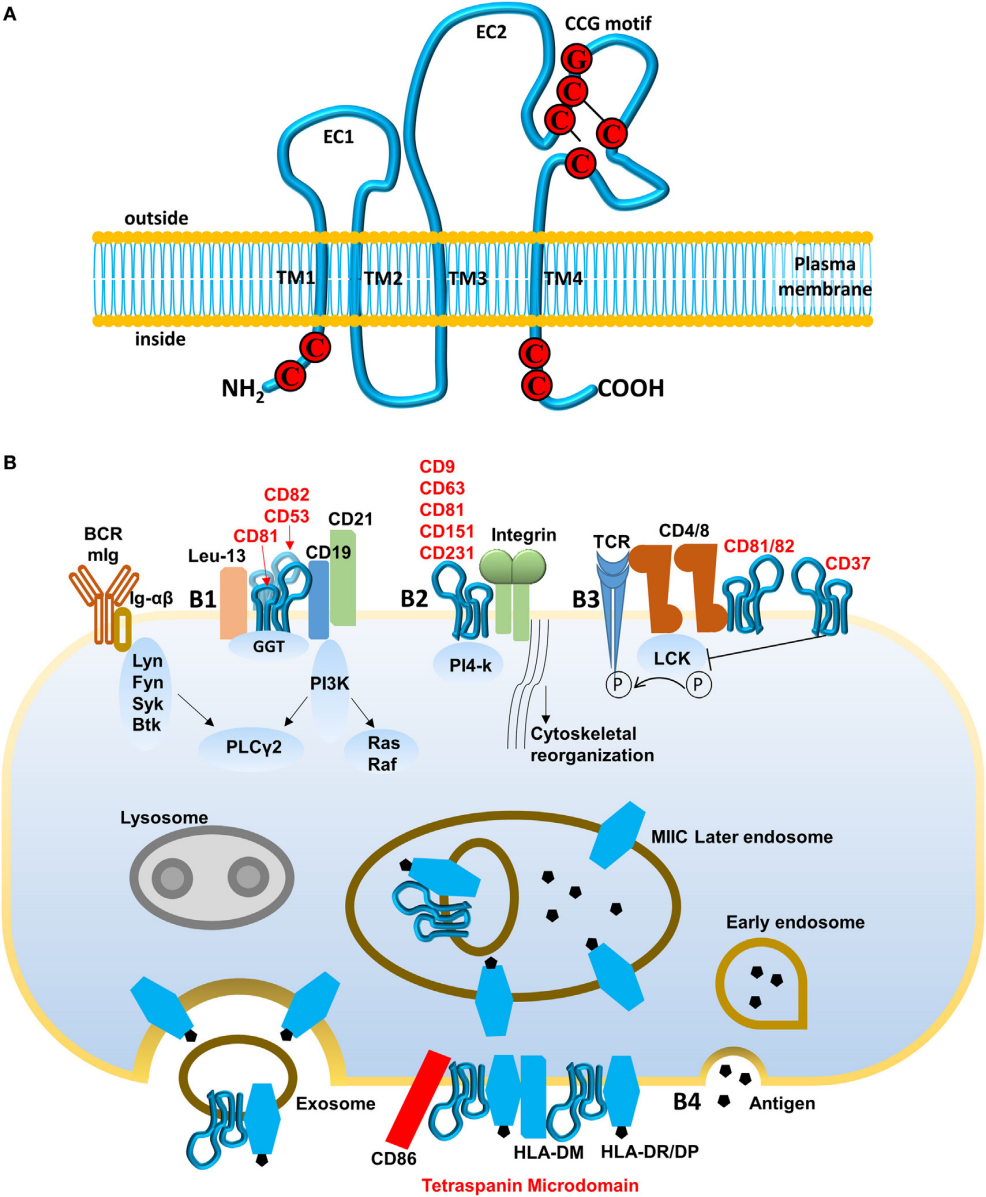
Structure of tetraspanin and pathways regulated by tetraspanins. **(A)** Schematic diagram of tetraspanins. Tetraspanins present four transmembrane domains (TMs) intracellular N- and C-termini and two extracellular domains (EC1 and EC2). CCG motif is formed with cysteine–cysteine–glycine (marked by red) and two disulfide bonds (marked by black line). **(B)** Pathways regulated by tetraspanins. (B1) B cell receptor (BCR) activation mediated by CD19–CD81–CD21 complex. Ig-α/β receive signals and are phosphorylated by Src kinase (Lyn, Fyn, or Btk), then recruit Syk kinase for initiating downstream signal pathway PLCγ2, Ras/Raf. Tetraspanin CD81, associated with CD52 and CD82, binds C19/CD21/Leu-13 signal-transducing complex and actives PLCγ2 through PI3K, which lowers the threshold for BCR signaling. (B2) Integrin-mediated cell adhesion. PI4-k, associated with various tetraspanins (CD9, CD63, CD81, CD151, and CD231), interacts with and promotes integrins to modulate cell spread and migration. (B3) T cell-B cell contact (TCR) pathway mediated by tetraspanins CD81, CD82, and CD37. CD4 and CD8 associate with Lck kinase to activate TCR signaling but their interaction with CD81, CD82, and CD37 interferes with phosphorylation of Lck kinase and may inhibit TCR signaling. (B4) Endocytic pathway for antigen presentation. Recognized antigens are internalized, processed, and loaded onto MHC class II molecules during the late endosome stage. Major histocompatibility complex class II mediates transport to the cell surface and the release of exosomes. Tetraspanin microdomains in antigen-presenting cell membranes are enriched for specific peptide–MHC class II complexes, peptide editor human leukocyte antigen-DM, and CD86 among other proteins. This selecting domain probably facilitates antigen presentation and T-cell activation, increasing MHC avidity.

Tetraspanins belong to a protein family in which members contain intracellular N- and C-termini, two extracellular domains (EC1 and EC2), and specifically four transmembrane domains (Figure 3A; 6, 7). Each phylum has evolved its own particular tetraspanins with distinction in the variety and abundance in different species. Despite this, the chemical composition of tetraspanins is highly conserved among species with four or more cysteine residues in a highly conserved “CCG” motif in the EC2 domain (8). There are 33 tetraspanins found in humans (Tables 1 and 2) and most of them preserve the characteristics of the ancient sequence in domain EC2.

**Table 1:** The regulation and function of tetraspanins and their interacting partners.

Protein	Regulate^a^	Regulated by^a^	Binds^a^	Role in cell^a^
TSPAN1	N/A	TP73, mir-8	NFKBIB	Endocytosis by proliferation

TSPAN2	CTNNB1, Jnk, BAX	TCF7L2, calmodulin, ERN1, RTN4, TGFB3, dexamethasone, d-glucose	SNX13, MTCH1, REEP6, PTGFRN, ARF6, GLP1R, LPCAT3, ZDHHC6, LGALS3, TSPAN3, DAGLB, LCLAT1, HSDL1, FAM241A, SNX14	Myelination, degeneration, development, differentiation, formation, upregulation, activation in, apoptosis

TSPAN3	N/A	FAS, neutrophils, PAX3, camptothecin, L-dopa, mir-197	ITGB1, LPAR1, RNF13, GABARAP, RDH14, MAP1LC3B2, SNX17, UGCG, FAM189B, GOLGA7, ZDHHC9, TNFRSF10B, RNF149, STX6, CDC6	Migration, proliferation

TSPAN4	Protein–protein complex	PLAG1, HK cells, MGEA5, hydrogen peroxide, CLOCK, estrogen	ITGA3, ITGB1, ITGA6, LOC100996763/NOTCH2NL, CREB3, Ul94, CD81, CD9, CLDN11, peptide, miR-1-3p (and other miRNAs w/seed GGAAUGU)	N/A

TSPAN5	N/A	CST5, WT1, MGEA5, TNFSF11, STAT4, beta-estradiol	ATP2B3, LCLAT1, RDH14, FAM210B, KLHL2, THAP11, TMEM87A, PIEZO1, NAT14, BSCL2, AGPAT3, TVP23C, ALG11, SOAT1, SNX25	Adhesion, proliferation, osteoclastogenesis

TSPAN6	N/A	Seocalcitol, SOX4, RBM5, HRAS, TP53, SNX27, retromer	EVA1C, TMEM185A, CLEC5A, TNFRSF17, ASIC4, CDS1, TMEM30B, VNN2, SERPINA12, LYPD4, GPR141, LRRTM1, MAVS, TMEM173, IFIH1	N/A

TSPAN7	N/A	MYC, EZH2, HOXD3, IL15, LMO1, TAL1, NEUROG1, HDAC4, influenza A virus [A/Bangkok/RX73(H3N2)], CD3, PAX3, omeprazole, LIF, NKX2-1, large T antigen	HAVCR2, PI4KA, CREB3, BBS1, NEF, ADCY5, CACNA1A, KPTN, RBL1, LGALS3	Shape change, cell spreading

TSPAN8	GCG	FGF10, TCF, STAT5A, CBX5, CTNNB1, 2-bromoethylamine, SMARCA4, AR, doxorubicin, indomethacin, captopril, hexachlorobenzene, cyclophosphamide, lomustine, puromycin aminonucleoside	ITGB1, ITGA3, EPCAM, integrin, ITGA6, CLDN7, ACTA1, ATP1A1, integrin alpha 6 beta 1, integrin alpha6 beta1, CD44, PDX1, miR-125b-5p (and other miRNAs w/seed CCCUGAG)	Cell movement

TSPAN9	N/A	EAhy926 cells, tamoxifen, ESR1, dexamethasone, cyclosporin A, NEF, CD3	ELAVL1	N/A

TSPAN10	N/A	PRDM1	HSD17B13, ADAM10, ADGRG5, PNLDC1	N/A

TSPAN11	N/A	N/A	POMC, CYB5R3, IGLL1/IGLL5, TM9SF4, ITGA7, ITGA6, REEP5, NRP2, ESYT1, ARL6IP5, ITGB1	N/A

TSPAN12	ADAM10, APP	GATA2, MGEA5, CLDN7, UPF2	FTT0715, TFCP2, LRP5, NDP, FZD4, TSPAN12, ADAM10	Proteolysis in, maturation in

TSPAN13	Cyclic AMP	CBX5, STAT5A, SMARCA4, PMSG, ESR2, HDAC4, TNFSF11, TGFB3, Cg, UPF2	GAG, GLP1R, APP, ELAVL1	Osteoclastogenesis, accumulation in

TSPAN14	GP6	Tretinoin, TGM2	ADAM10, PIK3R2, DPY30, PIK3CA, PIK3R3, ATP13A2, ELAVL1, HNF4A, REST	Molecular cleavage in

TSPAN15	CDH2	TCF7L2, F2RL1	P2RY12, AGTR1, RETREG3, LPAR6, ADAM10, SLC7A1, SYPL2, SLC22A16, IPPK, FZD10, C3AR1, HTR3A, GYPB, ADGRE5, CLCC1	Molecular cleavage in

TSPAN16	N/A	N/A	N/A	N/A

TSPAN17	N/A	KLF3, SATB1, calmodulin	PRAF2, FAM210B, ATP2A3, DHRS7, CCDC115, FAM189B, AGPAT3, TYW1, GHDC, PNPLA6, SLC44A1, RNF149, RETREG3, EPHX1, GP1BB	N/A

TSPAN18	N/A	2-amino-5-phosphonovaleric acid	FITM2, RNF130, iucD	N/A

TSPAN19	N/A	N/A	N/A	N/A

UPK1B	N/A	UPK3A, UPK2, CNR1, TP63, PD 153035, rosiglitazone, troglitazone	UPK3A, SNX31, BCL2L13, BNIP2, CCDC155	Differentiation

UPK1A	N/A	TP63, ATG16L1, OSM, SLC13A1, Caco2 cells, troglitazone, PD 153035	ECEL1, DIRC2, TMEM223, TMEM62, LMF2, TUSC3, SOAT1, FZD3, DPY19L1, TMEM39A, FZD1, LRRC8A, NAT14, PIGO, CIB1	Differentiation

PRPH2	RHO, ROM1, 26s proteasome	CRX, RHO	PRPH2, ROM1	Quantity, formation, function, synaptic transmission, overload in, length, generation, cell death, morphology

ROM1	N/A	AHI1, PLAG1, PRPH2, NRL, NRG2, influenza A virus [A/Bangkok/RX73(H3N2)], NRG1, dihydrotestosterone, EGF	PRPH2, SPTLC2, PHGDH, ITSN1, EPN1	Electrophysiology, degeneration, abnormal morphology, length, synaptic transmission, apoptosis, size

CD151	ITGA3, ITGB1, ERK1/2, PRKCA, PTK2, P38 MAPK, PI4KA, Akt, CD63, PRKCB, collagen type I, CD81, CD151, laminin (complex), FN1	MYC, ZDHHC2, PAX3, RET, integrin alpha 6 beta 4, mir-193, MITF, SMARCD3, T, BCL6, MKL2, MKL1, MGEA5, MYL2, valproic acid	ITGB1, ITGA3, ITGA6, ITGB4, CD9, ITGB3, ITGA5, GRAMD1C, integrin alpha 3 beta 1, TMED10, PI4KA, CD81, CD63, PRKCB, TMPRSS11B	Migration, adhesion, proliferation, abnormal morphology, thickness, lack, effacement, morphology, activation in, cell spreading

CD53	BCL2L1, BAX, DHX32, KRT20, GLS, TPPP3, TFDP1, MRPL32, FAM43A, CD53, PRKCA, PRKCB, caspase, Akt	Tretinoin, 17-alpha-ethinylestradiol, DYSF, CD3, RARA, IL15, RUNX1T1, RUNX1, MGEA5, SOX4, CREBBP, EP300, paclitaxel, PRDM1, vitamin E	PRKCA, PRKCB, ITGB1, CD81, GGT1, CD82, ITGA4, CD37, CD2, CD9, miR-224-5p (miRNAs w/seed AAGUCAC)	Apoptosis, invasiveness

CD37	IgG1, Immunoglobulin, IgG, CD4, LCK, CD8, IgM, IGHG1, adenosine, Lfa-1, RAC1, ICAM1, IL2	IL13, IGF1R, fluvoxamine, lipopolysaccharide, B lymphocytes, plasma cells, RAF1, PD98059	ACPA, PURL, YBTQ, PG8786 084, CD19, CD53, SYK, KARS, PTPN6, LYN, PIK3CD, PIK3CG, CD81, MHC class II (complex), CR2	Proliferation, adhesion, activation, chemotaxis, transendothelial migration, recruitment, cell death, cell division, internalization by, activation in

CD82	CD82, EGFR, BCAR1, MET, PRKCA, RAC1, CRK, focal adhesion kinase, GRB2, ITGA3, CANX, ITGA5, ITGB1, PRKCB, SHC1	IL1B, CD82, NFKBIA, ERBB2, APP, APBB1, IL6, TP63, NFkB (complex), ZFPM1, GSK3B, AURKB, P38 MAPK, mir-15, NEUROG1	CD81, CD19, CD9, ITGA3, ITGB1, ITGA6, EGFR, PRKCA, MET, NFKB1, CD1D, PRKCB, ITGA5, CREB3, integrin beta 1	Migration, invasion, transcription in, adhesion, accumulation in, motility, anoikis, invasion by, differentiation, signaling in

CD81	CD19, IFNG, MMP14, TNF, CD81, IgM, IgA, ERK1/2, PRKCA, IgG1, IgG, Igg3, dopamine, SP1, GTF3A	CD81, phorbol myristate acetate, hepatitis C virus JFH-1, LY9, WIPF1, curcumin, HIST1H1T, Hist1h1a, hydrogen peroxide, butyric acid, ZBTB16, HRAS, laminin 5, interferon alpha, ADORA2A	E2, CR2, ITGB1, CD19, ITGA3, CD9, CD82, IGSF8, PTGFRN, CLDN1, ITGA5, HNRNPD, RAC2, E1, CD151	Proliferation, abnormal morphology, adhesion, migration, motility, differentiation, number, phosphorylation in, entrance, binding

CD9	CD9, IL2, ITGA3, ITGA5, PRKCA, ITGB1, SRC (family), MMP9, CBL, ERK1/2, CD69, YAP1, DPP4, CASP3, ERVW-1	Decitabine, trichostatin A, forskolin, CD9, ZDHHC2, BCAP31, PRDX1, FOLR1, methylprednisolone, lactacystin, CCR5, MYC, MYCT1, E2F1, CXCR4	ITGB1, ITGA3, IGSF8, ITGA5, CD81, PTGFRN, CD151, ITGA6, ITGB3, CD82, ITGA2, ITGB4, Psg18 (includes others), PRKCA, CD36	Fusion, adhesion, proliferation, aggregation, migration, binding, apoptosis, motility, accumulation in, fertilization

CD63	KDR, PLC gamma, SRC, PTK2, ERK1/2, Akt, VTN, laminin (family), FN1, collagen, ITGB1, TNF	F2, cytochalasin B, IL3, IFNG, collagen(s), C5, IL5, CSF2, guanosine triphosphate, LEP, roscovitine, CDK5R1, AP3B1, NEUROG3, ZFPM1	ITGB1, LGALS8, RETREG3, LGALS3, LGALS9, TIMP1, ITGB3, ITGA3, PI4KA, CD151, MHC class II (complex), AP3M1, TSPAN2, TSPAN3, RNF13	Adhesion, differentiation, endocytosis by, internalization in, tubulation by, sprouting in, phosphorylation in, tyrosine phosphorylation in, migration, growth

TSPAN31	TSPAN31	Benzo(a)pyrene, TSPAN31, IRF4, CREBBP, EP300, heavy metal, TFAP4, EAhy926 cells	ELAVL1	Proliferation

TSPAN32	IL2	HOXA3, GATA2	N/A	Proliferation, organization, activation, aggregation

TSPAN33	PTGS2, NOTCH1, IFNB1, NOS1, NFkB (complex), ADAM10, Notch	NOTCH1, NOTCH2, *Mycobacterium tuberculosis* H37Rv, MAP3K8, tretinoin, IFNG, TLR4, TLR2, TLR3, dexamethasone	PLEKHA7, MSN, PDZD11, ADAM10, EZR	number, abnormal morphology, quantity, maturation in, signaling in, expression in, erythropoiesis

*^a^Organized from the information of each gene collected from thousands of publications by Ingenuity Pathway Analysis (IPA) program. Please note that relationships for the proteins in the list of the “Regulate” and “Regulated by” may be not direct or supported directly by experiments although most of them are derived from experimental data mining by IPA from published papers*.

**Table 2:** Expression of tetraspanins on B cells.

Name	Gene synonyms	Subcellular location	Expression on human B cell (TPM)		
			
			CD38^**−**^ naïve B cell	Isotype switched memory B cell^a^	Memory B cell
TSPAN1	NET-1, TSPAN-1	Nucleoplasm, vesicles	0	0	0
TSPAN2	FLJ12082, TSN2, TSPAN-2	Nucleoplasm	0	0.8	0
TSPAN3	TM4-A, TM4SF8, TSPAN-3	Nucleoplasm, Golgi apparatus	26	15	28
TSPAN4	NAG-2, TETRASPAN, TM4SF7, TSPAN-4		0	0	0
TSPAN5	NET-4, TM4SF9, Tspan-5		2	1	1
TSPAN6	T245, TM4SF6, TSPAN-6	Cytosol	0	0	0
TSPAN7	A15, CD231, DXS1692E, MRX58, MXS1, TALLA-1, TM4SF2		0	0	0
TSPAN8	CO-029, TM4SF3	Nucleoplasm	0	0	0
TSPAN9	NET-5	Nucleoplasm, Golgi apparatus, cytosol	0	0	0
TSPAN10	OCSP		0.6	0	0
TSPAN11		Vesicles	0	0	0
TSPAN12	NET-2, TM4SF12	Vesicles, microtubules	0	0	0
TSPAN13	NET-6, TM4SF13	Nucleus	72	13	17
TSPAN14	DC-TM4F2, MGC11352, TM4SF14	Vesicles	4	2	3
TSPAN15	NET-7, TM4SF15	Nucleoplasm, cytosol	0	0	0
TSPAN16	TM-8, TM4-B, TM4SF16		0	0	0
TSPAN17	FBX23, FBXO23, TM4SF17	Nucleoplasm	2	2	2
TSPAN18	TSPAN		0	0	0
TSPAN19					
UPK1B	TSPAN20, UPK1		0.6	0	0
UPK1A	TSPAN21		0	0	0
PRPH2	CACD2, rd2, RDS, RP7, TSPAN22		0	0	0
ROM1	ROM, TSPAN23	Plasma membrane, cytosol	0.5	0	0.6
CD151	PETA-3, RAPH, SFA-1, TSPAN24		1	2	2
CD53	MOX44, TSPAN25		240	185	221
CD37	TSPAN26		183	73	117
CD82	IA4, KAI1, R2, ST6, TSPAN27	Vesicles	16	28	48
CD81	TAPA-1, TAPA1, TSPAN28	Plasma membrane	15	9	12
CD9	BA2, MIC3, MRP-1, P24, TSPAN29	Plasma membrane	8	1	0.5
CD63	ME491, MLA1, TSPAN30	Vesicles	6	7	13
TSPAN31	SAS		5	3	6
TSPAN32	PHEMX, TSSC6		6	1	2
TSPAN33 (9)	MGC50844, Penumbra	Microtubules	31	11	26

*The information of “Gene synonyms” and “subcellular location” is obtained from “The Human Protein Atlas” (https://www.proteinatlas.org/). TPM, transcripts per kilobase million. Except Tspan19, all tetraspanins are expressed respectively on at least one of B lymphoma cell lines. The results were obtained by searching the gene symbol plus “B cell” for Biological conditions on Expression Atlas (https://www.ebi.ac.uk/gxa/home). The expression of human tetraspanins is determined by the RNA-seq data generated by the Blueprint Consortium*.

^a^The original description in the consortium is “Class switch memory B cell.”

### General Interactions Among Tetraspanins and Their Partners

Tetraspanins act as scaffold proteins to anchor multiple proteins—including other tetraspanins, partners of tetraspanins, and other proteins—to one area of the cell membrane, and form a tetraspanin-enriched microdomain (TEM) or tetraspanins web (10, 11). A recent study with super resolution microscopy provided a close view of TEM and demonstrated that TEM is composed of individual nanoclusters (<120 nm). There are no more than 10 CD53 molecules in a single tetraspanin cluster of CD53. The study also evaluated the distances between the individual clusters, including CD53, CD37, CD81, CD82, and the tetraspanin partners such as CD19 and major histocompatibility complex class II (MHC II) (12). Based on the sensitivity and stringency to different detergents, the interactions of tetraspanins and partners in TEM were classified into three categories (13, 14). This model allows for dynamic and adaptable interactions between tetraspanins and other surface proteins based on a descriptive categorization without correlation to functionality in the living cell. A recent review proposed a new applaudable classification of tetraspanin interactions based on their function in the formation of TEM: interactions (a) necessary to maintain tetraspanin structure, (b) that support tetraspanin web formation, (c) that add functional partners to the web, and (d) that facilitate intracellular events (6).

Three hypothetical models could be postulated to decipher the ways that tetraspanin microdomains enhance or regulate cellular signals and exert effects on fundamental biological processes. One model is that tetraspanins be considered a transmembrane linker connecting and augmenting signal transduction between membrane partners and intracellular-signaling proteins (15). Another model could propose that tetraspanins are involved in gathering partner membrane proteins which subsequently result in increased avidity and/or enhanced interaction with their ligands (16). The third hypothesis is that tetraspanins function as regulators by sequestering partners from signal transduction (17) thus preventing inappropriate signals and responses in resting cells. Without favoring any of these models at the present time, we now direct our attention to signal transduction and/or regulation by tetraspanins in immune cells (Figure 3B).

### Interaction of Tetraspanin CD81 and Its Partners in B Cell Receptor (BCR) Activation Pathway

In B cells, tetraspanins CD81 interacts with the CD19/CD21 signal-transducing complex to lower the threshold for BCR signaling (Figure 3B1). The multiprotein complex BCR consists of two parts: membrane immunoglobulin (Ig) with integral membrane domain, and signal transduction moiety Ig-α/Ig-β (also known as CD79A/CD79B) heterodimer tethered by disulfide bridges (18). When antigen binds to Ig, Src family kinase-like Lyn phosphorylates immuno-receptor tyrosine-based activation motif residues on the cytoplasmic tails of Ig-α/Ig-β, sequentially recruiting and activating Syk and Btk kinases, then initiating downstream signaling cascades of the Ras–MAPK pathway and PLCγ2 (19). The CD19/CD21 complex is thought to augment BCR signaling by decreasing the signaling threshold for B cell activation (20). Indeed, the direct association of tetraspanin CD81 with CD19 as a part of the CD19/CD21/Leu-13 complex is critical for both assembly and localization of this complex (20) and CD81-deficient B cells have been found to have reduced expression of CD19 and impaired B cell signaling (21). Furthermore, probably through interaction of tetraspanins CD9, CD53, CD82 with CD81, the CD19/CD21 complex has an additional layer of control over B cell signal regulation through formation of a CD19/CD21 complex with additional functional proteins, such as glutathione and oxidative homeostasis-related enzyme γ-glutamyl transpeptidase GGT (22).

### Function of Tetraspanin/Integrin Complexes in Signaling Pathway for Cell Migration and Adhesion

Tetraspanins associating with and forming tetraspanin/kinase-integrin complexes are implicated in both leukocyte and cell–cell adhesion (Figure 3B2) by causing signal activation and cytoskeletal reorganization. In B cells, by enhancing tyrosine phosphorylation levels, tetraspanin CD9 promotes β1 integrin-dependent mobility (23). In addition, tetraspanins CD9 as well as CD63, CD81 have been documented to associate with both PI4-kinase and integrin α3β1 in lymphoid cell lines (24). Finally, tetraspanins also have been found to enhance the avidity of integrins for neutrophil motility and T cell–B cell contact (25).

### Function of Tetraspanin CD37 and Its Partners in T Cell–B Cell Contact (TCR) Activation Pathway

Tetraspanins are implicated in TCR-induced activation and proliferation (Figure 3B3). Interaction of peptide with the MHC activates the TCR and initiates the downstream signaling cascade of Src kinases Fyn and Lck. Lck subsequently activates the functional proteins involved in T cell activation and proliferation. Interaction of Lck with CD4/CD8 plays crucial roles in this pathway (20); should CD4 associate with tetraspanins CD81/82 then Lck is sequestered from the TCR signaling pathway (26). Additional evidence shows that tetraspanin CD37 is coupled to TCR signal transduction mostly by influencing the dynamics of CD4-Lck distribution to TCR signal associated microdomains (27). Thus, tetraspanins regulate the T cell biologic process by influencing the TCR-CD4/CD8 cascade proximal to Lck mobilization.

### Functions of Tetraspanins and Their Partners in Antigen-Presenting Processes

MHC avidity and facilitation of T cell activation is also mediated by tetraspanins (Figure 3B4). Tetraspanins function in antigen-presenting cells (APCs) to assist in the presentation of the MHC–peptide complex to T cells. Tetraspanins CD81, CD37, CD82, CD53, and CD63, tether with MHC and associate with stimulators on exosome vesicles which are MHC II-enriched compartments. After the cell membrane is fused with MHC, the exosomes are released and can act as stimuli for T-cell proliferation (28). But there is an additional way in which tetraspanins work with MHC. Tetraspanin microdomains are enriched for MHC II, CD86, and the class II editor human leukocyte antigen in the membrane of APCs. This complex is referred to as the “CDw78^+^ microdomain” involved in T cell activation (20).

Through the above-enumerated regulatory pathways, the TEMs form a web for signal transduction from extracellular stimuli to intracellular-signaling components and ultimately regulate multiple biological processes, including cell activation, proliferation, adhesion, migration, and communication, as well as involvement in pathological conditions, such as autoimmune diseases, metastasis, and viral infection (Table S2 in Supplementary Material).

## Expression Profiles of Tetraspanins and their Partners on B Cells

Uniquely expressed molecules in certain B cell subsets may serve as markers of the subset or have special function for that particular subpopulation. Systematic analysis of expression of tetraspanins and partners of tetraspanins on B cells may facilitate an understanding of their biological involvement in B cell biology including B cell development and function.

### Expression of Tetraspanins on the Surface of B Cells

**Figure 4:**
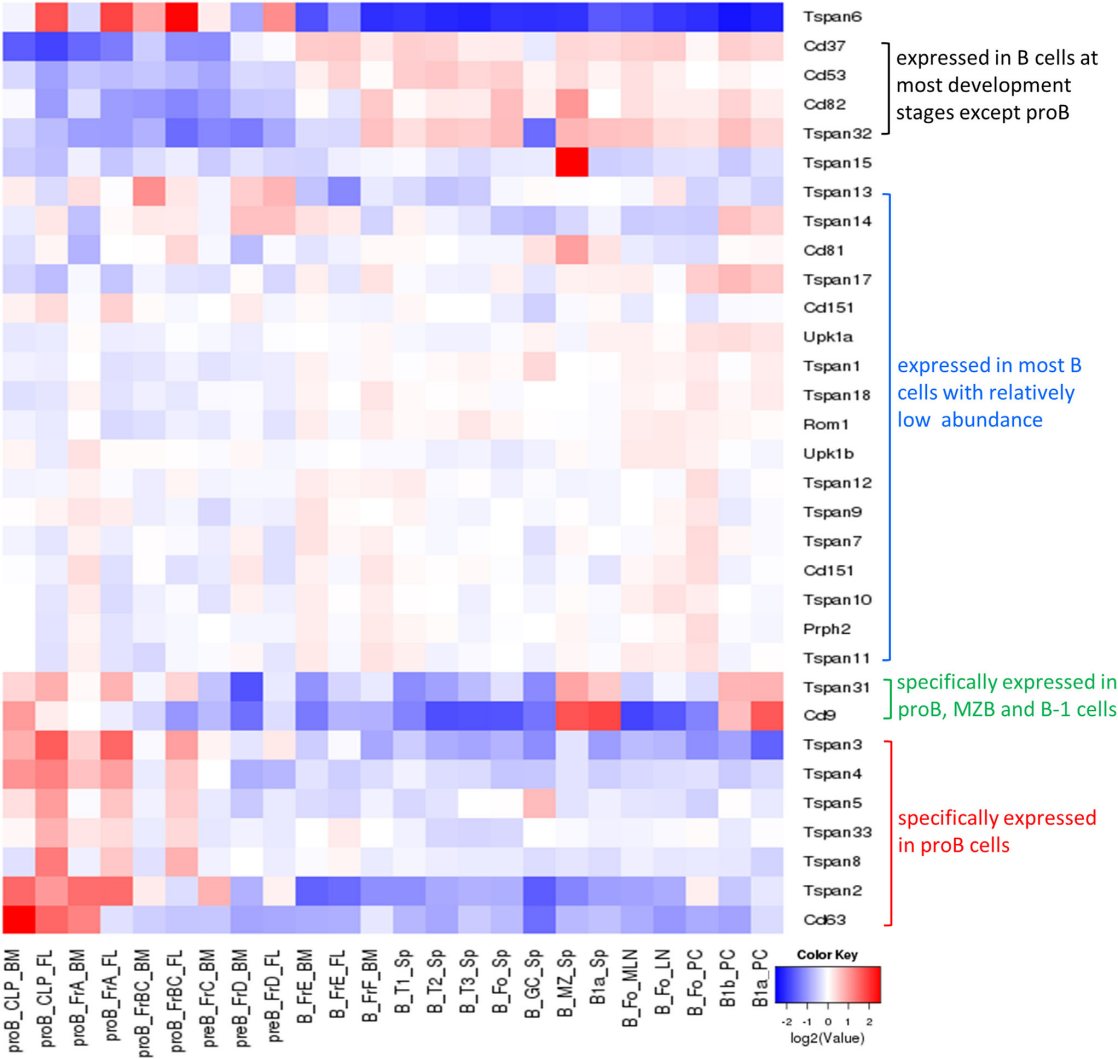
Expression of tetraspanins in murine B cell subsets. The heat map was obtained from http://www.immgen.org/by inputting the list of tetraspanins in “My GeneSet” and choosing B cells as the populations of interest. The gene expression level is determined by Affymetrix microarrays (GEO: GSE15907).

Most tetraspanins are expressed on B cells but differ in abundance in various B cell subsets at different developmental stages (Figure 4). mRNA transcripts of Tspan2-8, 31, 33, CD9, and CD63 are expressed at high levels in mouse progenitor B cells in the bone marrow but at very low levels, except for CD9 and Tspan31, in other B cell subsets (which mainly exist in periphery lymphoid organs). In contradistinction, CD37, CD53, CD82, and Tspan32 all show similar expression patterns of low level expression in mouse pro-B cells but high level in other B cell subpopulations. On tested human B cells (CD38^−^ naïve B cells, isotype switch memory B cells, and memory B cells), TSPAN3, TSPAN13, CD53, CD37, CD82, CD81, CD63, and TSPAN33 show relatively high levels of mRNA (TPM > 10). TSPAN2, 5, 10, 14, 17, 31, and 32, and UPK1B, ROM1, CD151, and CD9 have detectable mRNA transcripts. But the remaining tetraspanins have no detectable mRNA. In addition, the expression of all tetraspanins except TSPAN19 is detectable in at least one strain of B cell lymphoma cell lines. More expression profiles of tetraspanins can be found in Figure 4 and Table 2.

### Expression of Tetraspanin Partners on the Surface of B Cells

**Figure 5:**
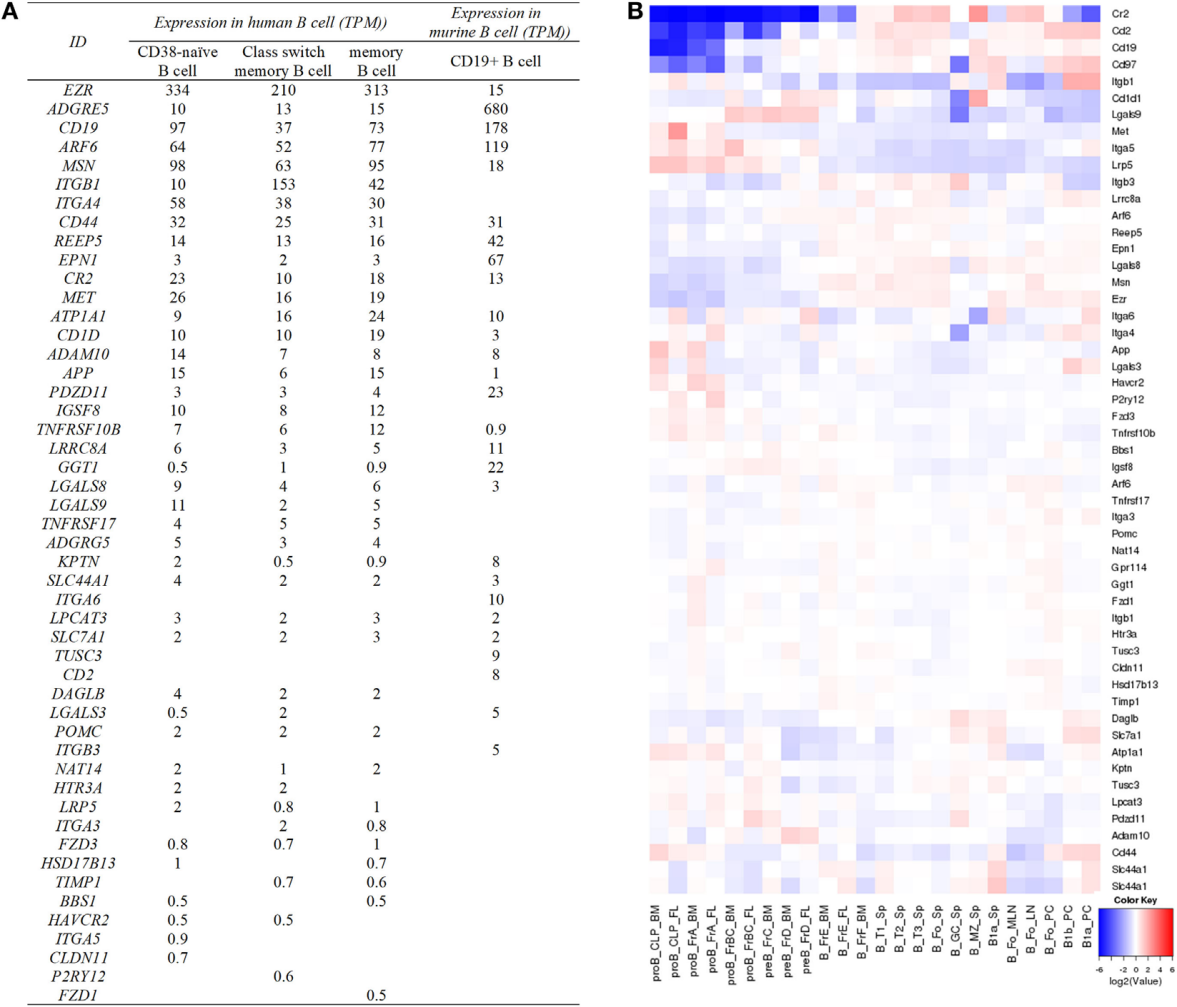
Expression of tetraspanin partners in human and murine B cell subsets. **(A)** The expression of cell surface partners of human and murine tetraspanins on specific B cells. The partners without TPM values are not listed in the table. Human data are determined by the RNA-seq data generated by the Blueprint Consortium, and murine data are from RNA-Seq CAGE (Cap Analysis of Gene Expression) analysis of mouse cells in RIKEN FANTOM5 project. **(B)** Expression of tetraspanin partners listed in **(A)** in murine B cell subsets. The heat map was obtained from http://www.immgen.org/ by inputting the list of cell surface partners of tetraspanin partners in “My GeneSet” and choosing B cells as the populations of interest. Based on the database, ADGRE5, ADGRG5, and CD1D in **(A)** are shown as Cd97, Gpr114, and Cd1d1 in **(B)**, respectively.

Affinity capture assays, protein-fragment complementation assays, and two-hybrid tests in the databases of BioGRID (Table S3 in Supplementary Material) and ingenuity pathway analysis (Table 1; Table S2 in Supplementary Material) have allowed for the identification of hundreds of tetraspanin interacting partners. The main cell surface partner proteins of tetraspanins are other tetraspanins, integrins, G-protein coupled receptors, and transmembrane receptors like CD19. After removal of the partners expressed in the cytoplasm and the nucleus, there are 93 membrane proteins which potentially interact with extracellular tetraspanins or tetraspanins on the same membrane (Table S3 in Supplementary Material). Some of the tetraspanins which interact with other tetraspanins include CD151, CD37, CD53, CD63, CD81, CD82, CD9, ROM1, TSPAN2, TSPAN3, and TSPAN12 (Table S3 in Supplementary Material). Most of the partners show high levels of expression in more than one mouse B cell subset (Figure 5). In tested human B cells (CD38^−^ naïve B cells, isotype switched memory B cells, and memory B cells), EZR, ADGRE5, ARF6, MSN, ITGB1, ITGA4, CD44, REEP5, EPN1, CR2, MET, ATP1A1, CD1D, ADAM10, APP, IGSF8, TNFRSF10B, and LGALS9 all show relatively high levels of mRNA (TPM > 10). More data can be found in Figure 5.

## Functions of Tetraspanins in B Cells

**Figure 6:**
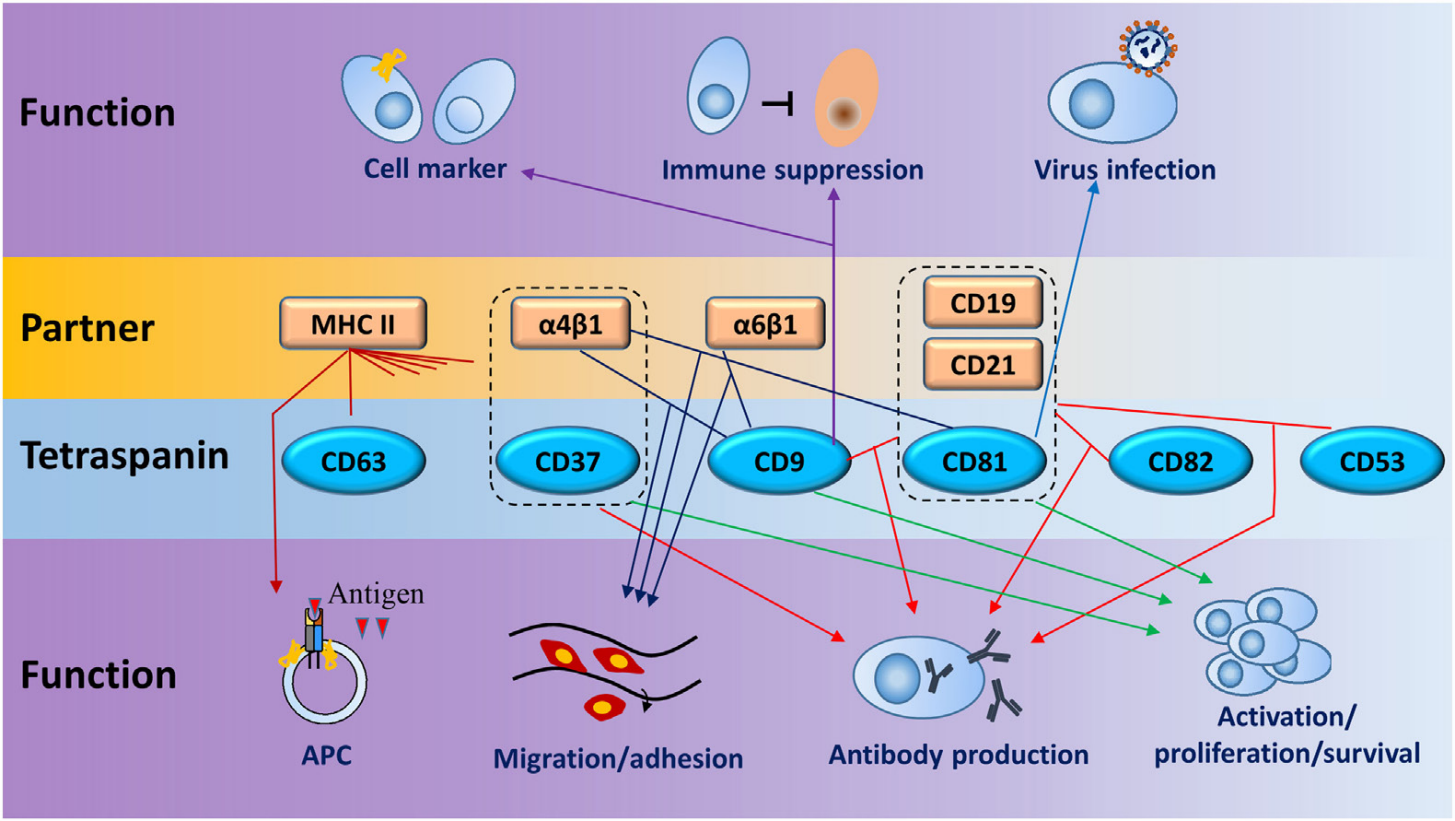
Schematic diagram for main functions of tetraspanins and their partners in B cells. Dotted lines enclose coreceptors formed by tetraspanins and partners. Lines between tetraspanins and partners or coreceptors indicate the interactions which have functions in B cells (line with arrow) in seven biologic processes, such as cell activation, antigen presentation, and antibody production. One tetraspanin or partner can have one or more functions on B cells. Same colored lines with arrows point to similar function or regulation.

Tetraspanins modulate cell adhesion, migration, and invasion which are strongly involved in cancer development and progression (29). The association between tetraspanin expression and cancer prognostic is found in many kinds of cancers (Table 1). In B lymphoma, aberrant expression of CD9, CD81, and CD82 was linked to B-acute lymphoblastic leukemia (30–32). Increased CD37 expression was found in B cell malignancies and thus CD37 antibodies were developed to deplete malignant B cells for the treatment of chronic lymphocytic leukemia (33). The correlation of tumorigenesis and tetraspanins is discussed in another submission for this topic, so here we focus on the functions of tetraspanins and their partners on normal B cell biology without any further discussion of B cell malignancy. Functions of tetraspanins are summarized in Figure 6 and Table 3.

**Table 3:** Function of tetraspanin on B cells.

Tetraspanin^a^	Keywords	Details of function on B cells
CD9	Markers, immune suppression, virus infection, activation, differentiation	Marker for murine marginal zone B cells, B-1, and plasma cells (34); a marker for plasma cell precursors in human germinal centers (35); novel cell surface marker of murine B10 cells and their progenitors (36); contributes to B cell activation and differentiation (37), and the survival of human GC B cells (38); involved in normalizing TH2- and TH17-driven airway inflammation in an IL-10-dependent manner (39); promotes inhibition of Th1-mediated contact hypersensitivity (36); enhances numbers of extracellular vesicles and improves the speed and efficiency of lentiviral gene delivery into T and B cells (40); Engagement of CD9 induces CD19 tyrosine phosphorylation (37)

CD81	Forms CD81–CD19–CD21 complex, proliferation	Controls lymphocyte homeostasis by facilitating the interaction with follicular dendritic cells through the VLA4/VCAM-1 axis (21); may interact with a putative ligand on a subpopulation of T cells to signal IL-4 production (41); determines CD19 membrane expression (42); interacts with CD19/CD21 complex and tetraspanins such as CD9, CD53, CD63, and CD82 to enable formation of tetraspanin-enriched microdomains (TEMs) (43); entry factor of hepatitis C virus (44)

CD53	Activation, adhesion, development, apoptosis, antibody production	Interferes with lymphocyte activation and cell adhesion; a direct genetic target for EBF1 which is a critical transcription factor for early B lymphocyte development, and can be induced by ectopic expression of EBF-1 (45); CD53 mediates PKCβ recruitment from cytosol to TEMs for B cell receptor activation (46). CD53 engagement with antibody against CD53 and Ig promotes activation of resting B cells into the G1 phase and induces Ig production in the presence of T cell supernatant (47)

CD63	Exosome production, virus infection, migration	Suppressor of exosome production and could regulate the exosome-mediated major histocompatibility complex class II-dependent T-cell stimulation (48); sensitized to and controls latent membrane protein 1-mediated NFkB activation for EBV persistence (49); cell migration by affecting the abundance of CXCR4 on the cell surface through IL-21-induced endocytosis and CD63-mediated endosomal recruitment (50)

CD37	Apoptosis, survival, antibody production	Regulates the membrane distribution of α(4)β(1) integrin crucial for activating the Akt survival pathways, increases apoptosis of plasma cells in germinal centers (51); initiates a cascade of events leading to apoptosis, counteracts death signals by regulating PI3K-dependent survival (52); promotes IgG1 production while inhibiting IgA immune responses *in vivo* and protects against the development of IgA nephropathy (53); control suppressor of cytokine signaling 3 (54)

CD82	Protection from cytotoxicity	Interferes with the capacity of the MHC-I complex to protect targets from NK-mediated cytotoxicity (55)

^a^Only list the tetraspanins discussed in Section “Functions of tetraspanins on B cells.”

### Act as Markers Identifying B Cell Subsets

As discussed above (Figure 4; Table 2), since some tetraspanins are enriched in specific B cell subsets, they may be used as markers to identify B cell subsets or diagnostic markers for certain diseases. CD9 is reported to be a special shared marker by B-1 cells, MZB cells, and plasma cells in mice. A study demonstrated that CD9 is expressed by plasma cells in response to antigens independent or dependent on T cells, suggesting CD9 is acquired by T cell dependent plasma cells (34). Another study demonstrated that CD9 is a cell surface marker for precursors of human plasma cells in germinal centers. It is based on the evidence that (1) compared to CD9^−^ cells, CD9^+^ B cells show higher Blimp-1 but lower Bcl-6 and Pax-5 protein levels, and a faster process of differentiation into plasmablasts in the presence of PC-generating cytokines; (2) expression of CD9 was induced and gradually increased in CD9^−^ GC-B cells under PC generating condition (35). A recent study showed that murine CD9 is a unique cell surface marker identifying IL-10 competent Bregs and their progenitors (36).

### Roles in Cell Activation, Proliferation, Survival, and Development

The CD21/CD19/CD81 complex modulates signal transduction events pivotal for development of B lymphocyte and the normal function of humoral immunity. As shown in Figure 1, CD19, a hallmark of B cells, is sustained in its presence on B cells from the earliest pro-B cell stage to plasmablasts during development. CD19 functions as a co receptor of B cells in association with CD21 and CD81. In the complex, CD19 is the signaling molecule bound by Src-family kinases and PI-3 kinase, CD21 binds opsonized foreign antigens, and CD81 is associated with other tetraspanins including CD82, CD63, CD53, and CD9 to enable formation of TEMs (43). CoIP experiments demonstrated association of CD9, CD81, and CD82 with CD19 and digitonin treatment disrupted the CD9/CD19 and CD9/CD81 associations but not the CD81/CD19 association, implying that the association of CD9 with CD19 is through CD81 instead of another molecule. Different proteins including CD19 could be tyrosine phosphorylated which is induced by engagement of CD9, suggesting that CD9 involves B cell activation and differentiation (37). CD81 is one of the key proteins participating in controlling homeostasis of lymphocytes through modulating their proliferation. CD81 KO mice show reduced B1 cells and CD19 expression on B cells although the development of T cells and conventional B cells is normal. Moreover, in CD81 KO mice, the proliferative response of T cells is enhanced following TCR engagement, while proliferation of B cell responding to BCR cross-linking is severely impaired (21). Engagement of CD81 with hepatitis C virus (HCV) envelope protein E2 could protect B cells from apoptosis (56), and induce B cell activation (57, 58) and V_H_ hypermutation (59).

CD9 appears to function in B cell activation and differentiation based on its expression in specific B cell subsets and its functional interaction with CD19. CD9 is reported as a cell surface marker of B-1 B cells, MZ B cells, and plasma cells, but the development of these B cell subsets as well as the humoral immune responses to antigens appear to be normal when CD9 is knocked out (60). A recent study also confirmed that most of the tested markers expressed on total B cells are not significantly altered when CD9 is mutated. Interestingly, however, the frequency of occurrence of IL10 competent Breg (B10 cells) is increased and CD23 expression is reduced on B10 cells when CD9 is knocked out (36). Moreover, CD9 is reported to facilitate interacting with human follicular dendritic cells through the VLA4/VCAM-1 axis and contribute to the survival of germinal center B cells (38).

In addition to its involvement in Ig production, CD53 also contributes to B cell differentiation. A study demonstrated that CD53 is a direct genetic target of EBF-1, a critical transcription factor in early B lymphocyte development. CD53 has functional binding sites for EBF-1 in its promoter elements and can be induced by ectopic expression of EBF-1 (45). CD53 transcripts are enhanced significantly by mitogenic stimulation, implying that CD53 may be involved in the transport of signals important for cell proliferation. Under conditions of serum deprivation, ligation of CD53 in B lymphoma cells triggers an AKT-mediated survival response and prompts a significant reduction in caspase activation and the number of cells that enter apoptosis (61). By using live-cell imaging and gene knockout mice, a recent study demonstrated that CD53 is specifically required for the recruitment of PKCβ (the protein kinase C family member) from cytosol to CD53-enriched TEMs on the plasma membrane to activate PKCβ for antigen-dependent BCR activation, suggesting that TEMs act as signaling hotspots (46).

The tetraspanin CD37 has profound roles in B cell proliferation and survival. CD37 regulates the plasma membrane distribution of α(4)β(1) integrins by controlling their mobility and clustering, a necessary step in activating Akt survival pathways. It is reported that the number of IgG-secreting plasma cells is reduced in lymphoid organs when CD37 is knocked out in mice, possibly due to the impaired association of VCAM-1 to the α(4)β(1) integrin for the Akt survival pathway with the corollary of increased apoptosis of plasma cells in germinal centers (51). In a recent study, CD37 knockout in mice can drive B cell lymphoma progression through constitutive activation of the IL6 pathway by losing the control of suppressor of cytokine signaling 3 (54). Although CD37 is crucial for B cells to survive and provide long lasting immune protection, another study reported that CD37 may trigger a cascade of events resulting in apoptosis when it is tyrosine phosphorylated and binds with signaling factors. The study also found that CD37 mediates SHP1-dependent death *via* its N-terminal domain, whereas it antagonizes death signals through the C-terminal domain by mediating PI3K-dependent survival (52).

CD82 associates with MHC-I at the cell surface of B cells and could interfere with the capacity of the MHC-I complex to protect targets from NK-mediated cytotoxicity (55). CD63 is reported as a suppressor of exosome production and could regulate exosome-mediated MHC II-dependent T-cell stimulation (48).

### Roles in Antibody Production

In addition to its role in B cell proliferation and selection of IgG^+^ plasma cells, CD37 promotes IgG1 production while inhibiting IgA immune responses *in vivo*. CD37 deficiency causes a reduction of serum IgG1 levels and alters B cell responses to T cell-dependent antigen under suboptimal costimulatory conditions (62). Besides the reduction in serum IgG1 levels, CD37 deficiency in B cells causes high levels of IL-6 and is directly responsible for the increased IgA^+^ plasma cell numbers and IgA production levels in CD37^−/−^ mice. CD37^−/−^ mice are better protected from infection by *Candida albicans* than WT mice due to the increased *C. albicans*-specific IgA antibody levels. Neutralization of IL-6 *in vivo* could reverse the enhanced IgA response in CD37^−/−^ mice (63). Therefore, it is not surprising to find that CD37 protects against the development of IgA nephropathy by controlling the formation and deposit of IgA–antigen complexes in the glomerulus (53).

The absence of CD81 on murine B cells causes a defect of antibody responses to T cell-dependent protein antigens and reduces the production of IL-4 that is specific to antigens in both spleens and lymph nodes. A putative ligand on a subpopulation of B and T cells may interact with CD81 to signal IL-4 production (41). The function of CD81 was confirmed in a patient carrying a homozygous mutation of the CD81 gene which caused the syndrome of antibody deficiency by disrupting the CD19 complex in B cells and impairing BCR activation although the CD19 alleles in the patient are normal. Otherwise, the patient had neither overt T cell subset nor functional defects, similar to CD19-deficient patients. Further study revealed that CD19 membrane expression critically depends on CD81 and no cell surface CD19 could be observed on B cells from the patient who had the mutated CD81 (42).

Besides the above proteins, CD53 also plays an important role in activation and differentiation of B cells. CD53 engagement with both the MEM-53 antibody against CD53 and a polyclonal anti-mouse immunoglobulin promotes B cell activation from resting status into the G1 phase, and induces Ig production when treated with T cell supernatant (47).

### Immune Suppression

A study has shown that CD9 is a unique cell surface marker for murine B10 cells and their progenitor cells. Moreover, CD9^+^ B cells are capable of inhibiting contact hypersensitivity mediated by Th1 cells *in vivo*. Further *ex vivo* assays demonstrated that CD9 is involved in cross-talk between B cells and T cells, which is required for IL10^+^ B cells to suppress proliferation of T cells (36). Another study also indicated that IL-10^+^ Bregs are enriched in a CD9^+^ B cell subset and their homeostasis is altered by induced allergic asthma. Adoptive transfer of CD9^+^ B cells in asthmatic mice normalizes lung function in an IL-10-dependent manner *via* inhibiting inflammation driven by Th2 and Th17 cells (39).

### Roles in Virus Infection

Both CD9 and CD63 were identified and found to be transcribed by IgM^+^ cells in different tissues of rainbow trout (*Oncorhynchus mykiss*). And the abundance of CD9 transcripts decreased significantly in IgM^+^ splenocytes when the cells were exposed *in vitro* to viral hemorrhagic septicemia virus (64). Overexpression of CD9 caused a significantly higher yield of extracellular vesicles and improved the speed and efficiency of lentiviral gene delivery into T and B cells with the lentivirus produced in the CD9 high cells, although the virus titers were not increased. The study indicates an important role for CD9 in membrane fusion, virus infection, and information transfer mediated by extracellular microvesicles (40).

Viral oncogene latent membrane protein 1 (LMP1) accumulates within intraluminal vesicles to avoid degradation and thus constitutively activates NF-κB which is important for EBV persistence in B cells. CD63 associates with LMP1 and facilitates the inclusion of LMP1 into vesicles lacking MHC II. Preclusion of LMP1 assembly within CD63-enriched domains by C-terminal modifications of LMP1 leads to NF-κB overstimulation. Interference through shRNAs against CD63 causes redistribution of LMP1 and leads to a dramatic increase in LMP1-induced NF-κB activity, indicating that CD63 is sensitized to and controls LMP1-mediated NF-κB activation (49).

CD81 plays important roles in HCV infection by acting as a HCV entry factor (65), promoting HCV RNA replication (66), and reducing HCV-induced immune responses (44). B cells expressing CD81 can be infected by HCV and serve as reservoirs for chronic HCV infection (67).

### Cell Migration, Adhesion

CD63 plays important roles in cell migration as it can affect the abundance of CXCR4 on the cell surface through IL-21-stimulated endocytosis and endosomal recruitment. Restimulation of activated B cells with T cell-produced IL-21 accelerates CXCR4 internalization by inducing endocytosis-related GRK6 expression. The level of CD63 is strikingly elevated in activated Bcl6-deficient B cells and downregulation of CD63 mRNA with siRNAs upregulates CXCR4 expression on the B cells. Activated B cells treated with Bcl6 inhibitor have a similar phenotype to Bcl6-deficient B cells: increased CD63 mRNA expression and downregulated CXCR4 expression (50). It is reported that CD53 plays an important role in homotypic cell aggregation of lymphocytes and may interfere with lymphocyte activation and cell adhesion. HI29, an anti-CD53 monoclonal antibody, was able to induce homotypic cell aggregation in a B cell strain from a leukocyte adhesion deficiency patient. Moreover, pre-incubation with MEM53, another antibody against CD53, can block such aggregation but anti-CD44 or anti-CD49d mAbs have no blocking effects. Tetraspanins also interact with integrins which function within the area of cellular motility. Ectopic expression of CD9 has been reported to enhance B cell migration *via* interacting with integrins α6β1 and α4β1 (23). α4β1 on B cells can also be associated with CD81 (68).

## Functions of Tetraspanin Partners Expressed on B Cells

The partners of tetraspanins have multiple functions in B cells—including regulation of B cell activation, survival, development, antibody production, virus infection, and signal transduction—through mechanisms which may not be correlated with the interaction between tetraspanins and the partners. More details can be found in Table 4.

**Table 4:** Functions of tetraspanin partners on B cells.

Partner^a^	Tetraspanin interacted	Function of partners on B cells
Adam10	TSPAN33	Required for development of T1 B cells to marginal zone B cells (69); increased in allergic patients, sheddase of CD23, and promotes IgE production (70); release of TACI in B cells and reflects systemic and compartmentalized B cell accumulation and activation (71); required for CD23 sorting into B cell-derived exosomes (72)

CD19	CD37, CD82, CD81	Interacts with CD21, CD81, and B cell receptor (BCR) complex to augment signals by the pre-BCR/BCR for transducing signals; modulates B-cell fate decisions at multiple stages of development (37, 73, 74); pivotal for Akt activation that is mediated by BCR (75); intensifies Src-family PTK activation following BCR ligation (76); important for recruitment of Vav, Grb2, PI3K, phospholipase Cγ2, and c-Abl, or SHPI and SHIP phosphatases (77)

CD1d	CD82	Regulates interaction between activated T cells and B cells which is crucial to B cell proliferation and antibody production (78); mediates antigen presentation and augments antibody responses (79); CD1d knockout in mice impairs resistance to *Borrelia burgdorferi* infection due to impaired antibody production (80); CD1d upregulation on Breg cells is induced by chronic intestinal inflammatory conditions (81)

CD2	CD53	Expressed preferentially on fetal thymic B cells, anti-CD2 antibody increases IL-4-dependent Ig production by thymic B cells (82); the interaction of CD2 with LFA-3 enhances B cell responses (83); modulates T cell-dependent B cell activation (84); all peripheral B cell, the majority of bone marrow B cells and half of pre-B cells are CD2 positive (82).

CD36	CD9	Expressed by most resting MZ B cells, has no role in the development of B cells but regulates both primary and secondary phosphoryl choline antibody responses during *S. pneumoniae* infection (85); a target gene of POU2F2 transcription factor (86)

CD44	TSPAN8	Complex of CD44 and CD74 binds macrophage migration inhibitory factor to induce B cell survival (87); CD44 engagement could prevent polyclonal B cell activation by CD40L, while allowing B cell activation by interacting between soluble IgM and CD40L (88); required for interaction between B cells and monocytes independent of the B-cell receptor (89); induces murine B cell activation through hyaluronate-CD44 interactions (90)

CR2/CD21	CD37, CD81	An Epstein–Barr virus receptor on B cells and transduces signals (91); an interferon α receptor on B cells (92); a novel target for depletion of EBV-infected cells (93); binds to gp350 for efficient EBV infection of resting B cells (94); CD21^low^ B cells are apoptosis-prone (95); uncoupling of CD21 and CD19 significantly reduces survival of GC B cells and titers of secondary antibody (96); defective B cell ontogeny and humoral immune response is similar between human CD21 transgenic mice and aging wild-type mice (97); premature expression of human CD21 promotes B cell deletion and reduces auto-antibody titer significantly (98); CD21/CD19-mediated signaling enhances B cell survival in primary immune response (99); forms complex with CD19 and CD81 into signaling-active lipid rafts (100, 101)

MET	CD82	Recruited to CD74/CD44 complex and activated by HGF then leads to a survival cascade of B cells (102); stimulated by HGF/SF and enhances GC B cell adhesion to both VCAM-1 and fibronectin; predominantly expressed on CD38^+^CD77^+^ tonsillar B cells (103)

TNFRSF17/BCMA	TSPAN6	Reduced BCMA expression on peripheral B cells associates with severe syndrome of systemic lupus erythematosus (SLE) patients (104); preferentially expressed by autoantibody producing CD180^−^ B cells from active SLE patients (105); associates with TNF receptor-associated factors and activates NF-κB, elk-1, c-Jun N-terminal kinase, and p38 MAPK (106); receptor of a TNF homolog and implicated in SLE disease mediated by B cells (107)

*^a^Obtained by searching keywords in title with the partners of tetraspanins in Table 4 and keywords in title/abstract with “B cell,” and excludes the literature about cancer, lymphoma, and leukemia*.

## Therapy Strategies for Immune Diseases Correlated with Tetraspanins and their Partners

Most studies on employing tetraspanins and their partners as therapy target of diseases involve cancer treatments, a subject outside the scope of this review. For other diseases, there are some strategies using tetraspanins and their partners on B cells as therapy targets, as we explain below.

### Depletion of B Cell Subsets, Blockade of Receptors or Crosslinking With Antibodies Against Certain Tetraspanins or Partners

CD21 can be used as a target for depletion of EBV positive B cells as it is a receptor for EBV on B cells. CD19 is a hallmark of B cells and could be used as a target for B cell depletion in the treatment of autoimmune diseases, such as multiple sclerosis, rheumatoid arthritis, and systemic lupus erythematosus (SLE). There is a phase I clinical trial (Identifier: NCT00639834, ClinicalTrials.gov) using anti-CD19 antibody MDX-1342 together with methotrexate for the treatment of patients with rheumatoid arthritis.

CD81 is an entry factor for HCV infection. Monoclonal antibodies with high affinity to CD81 are generated for prevention of HCV infection (108).

TNFRSF17/BCMA is preferentially expressed in CD180^−^ B cells which produce autoantibodies and are significantly increased in SLE (104, 105, 109). TNFRSF17/BCMA and CD180^−^ B cell subsets would be ideal targets for SLE treatment.

CD44 engagement could control CD40L-mediated polyclonal B cell activation (88). Cross-linking of CD53 with antibodies against CD53 promotes activation of resting B cells, speeds up the entrance into the G1 phase of cell cycle, and induces Ig production during the incubation with T cell supernatant (47).

### Reduction of Protein Abundance With shRNAs or siRNAs Against Certain Tetraspanins or Partners

Interference through shRNAs against CD63 causes redistribution of LMP1, leads to a dramatic increase in LMP1-induced NF-κB activity, and would benefit treatment of EBV infection (49).

### Overexpression or Delivery of Certain Tetraspanins or Partners in B Cells

CD9 expression increases exosome production and promotes lentivirus infection (40), thus CD9 could be overexpressed in the engineered cells producing therapeutic exosomes to enhance the yield of exosomes and the delivery efficiency of exosomes.

### Interference of Tetraspanins or Partners With Small Molecules, Inhibitors, or Stimulators of Diseases

ADAM10 improves IgE production *via* its sheddase activity on CD23, an IgE receptor with low affinity (70). Adam10 increases in the B cells of allergic patients and Th2 prone mice (71) and would help diagnostically in predicting Th2 disease susceptibility. ADAM10 inhibitors could be used for attenuating allergic diseases.

### Immunotherapy With Certain B Cell Subsets Defined by Specific Tetraspanins or Partners

IL-10 secreting Breg defined by CD19 and CD9 in mouse (36) or CD19, CD27, and CD38 in human (4) could be enriched, expanded, and then adoptively transferred for treatments of autoimmune diseases.

## Author Contributions

FZ, XW, and XH summarized the literature, wrote the manuscript, and prepared figures. GR, SZ, and UB provided critical comments and wrote part of the manuscript. JS supervised all the work and wrote the manuscript.

## Conflict of Interest Statement

The authors declare that the research was conducted in the absence of any commercial or financial relationships that could be construed as a potential conflict of interest.
