# Supplementary material for: Expression and Function of Tetraspanins and Their Interacting Partners in B Cells
Source: Front Immunol. 2018 Jul 18;9:1606. doi: 10.3389/fimmu.2018.01606 (PMC6058033; doi:10.3389/fimmu.2018.01606)
Supplement: Supplementary file 1 [file table_1.DOCX]

| Protein | accession NO | Mus musculus* | | Gallus gallus | | Danio rerio | | Caenorhabditis elegans | | Drosophila melanogaster | | Aeromonas | |
| --- | --- | --- | --- | --- | --- | --- | --- | --- | --- | --- | --- | --- | --- |
|  |  | C | I | C | I | C | I | C | I | C | I | C | I |
| TSPAN1 | NP_005718.2 | 100 | 74 | 100 | 60 | 100 | 47 | 94 | 31 | 99 | 25 | 95 | 29 |
| TSPAN2 | NP_005716.2 | 100 | 83 | 100 | 55 | 100 | 48 | 96 | 27 | 96 | 23 | 97 | 24 |
| TSPAN3 | NP_005715.1 | 100 | 97 | 100 | 91 | 100 | 87 |  |  |  |  | 95 | 28 |
| TSPAN4 | NP_001020407.1 | 100 | 94 | 100 | 87 | 100 | 71 | 97 | 32 | 99 | 28 | 91 | 33 |
| TSPAN5 | NP_005714.2 | 100 | 100 | 100 | 99 | 99 | 91 | 96 | 45 | 97 | 46 | 93 | 31 |
| TSPAN6 | NP_00326.1 | 100 | 93 | 100 | 66 | 98 | 58 |  |  | 96 | 28 | 94 | 30 |
| TSPAN7 | NP_004604.2 | 100 | 98 | 100 | 93 | 100 | 84 |  |  |  |  | 97 | 31 |
| TSPAN8 | NP_004607.1 | 100 | 70 | 100 | 55 | 97 | 32 |  |  | 100 | 25 | 94 | 34 |
| TSPAN9 | NP_001161792.1 | 100 | 93 | 100 | 87 | 100 | 81 | 97 | 32 | 92 | 30 | 97 | 32 |
| TSPAN10 | NP_114151.3 | 86 | 64 | 69 | 52 | 69 | 43 | 72 | 32 | 76 | 37 |  |  |
| TSPAN11 | NP_001073978.1 | 100 | 87 | 100 | 57 | 99 | 71 |  |  | 92 | 31 | 93 | 34 |
| TSPAN12 | NP_036470.1 | 100 | 98 | 100 | 94 | 100 | 68 | 66 | 27 |  |  | 80 | 23 |
| TSPAN13 | NP_055214.1 | 100 | 96 | 100 | 88 | 100 | 67 |  |  | 99 | 46 |  |  |
| TSPAN14 | NP_001338198.1 | 100 | 97 | 100 | 85 | 99 | 72 | 95 | 38 | 98 | 43 | 91 | 29 |
| TSPAN15 | NP_036471.1 | 100 | 92 | 99 | 79 | 97 | 66 | 78 | 28 | 84 | 34 | 79 | 26 |
| TSPAN16 | NP_036598.1 | 92 | 30 | 77 | 34 | 88 | 35 |  |  | 79 | 26 | 89 | 26 |
| TSPAN17 | NP_036303.1 | 84 | 93 | 80 | 60 | 82 | 86 | 79 | 40 | 82 | 45 | 76 | 30 |
| TSPAN18 | NP_570139.3 | 100 | 91 | 100 | 81 | 100 | 69 |  |  | 95 | 28 |  |  |
| TSPAN19 | NP_001094387.1 | 96 | 27 | 60 | 37 | 96 | 26 | 88 | 23 | 79 | 22 | 68 | 25 |
| UPK1B | NP_008883.2 | 100 | 91 | 100 | 73 | 86 | 36 | 74 | 23 | 73 | 24 | 94 | 24 |
| UPK1A | NP_001268372.1 | 78 | 92 | 73 | 39 | 67 | 47 | 68 | 24 |  |  |  |  |
| PRPH2 | NP_000313.2 | 100 | 91 | 95 | 81 | 99 | 65 | 57 | 25 | 49 | 25 | 33 | 25 |
| ROM1 | NP_000318.1 | 94 | 85 | 90 | 35 | 96 | 39 | 30 | 26 | 38 | 28 | 31 | 26 |
| CD151 | NP_620598.1 | 100 | 94 | 100 | 79 | 100 | 71 | 93 | 32 | 97 | 29 | 94 | 35 |
| CD53 | NP_001035122.1 | 100 | 83 | 97 | 39 | 98 | 42 | 73 | 33 | 67 | 39 | 91 | 30 |
| CD37 | NP_001765.1 | 100 | 80 | 97 | 39 | 98 | 36 | 86 | 29 | 56 | 33 |  |  |
| CD82 | NP_002222.1 | 100 | 76 | 99 | 59 | 100 | 54 |  |  | 92 | 29 |  |  |
| CD81 | NP_004347.1 | 100 | 92 | 100 | 82 | 100 | 64 | 66 | 34 | 98 | 26 | 94 | 29 |
| CD9 | NP_001760.1 | 100 | 89 | 100 | 72 | 99 | 60 | 96 | 25 | 98 | 23 | 100 | 32 |
| CD63 | NP_001254627.1 | 100 | 79 | 100 | 51 | 100 | 46 | 98 | 33 | 97 | 28 | 100 | 50 |
| TSPAN31 | NP_005972.1 | 100 | 88 | 100 | 58 | 100 | 70 |  |  | 99 | 43 |  |  |
| TSPAN32 | NP_620591.3 | 79 | 64 | 73 | 46 |  |  |  |  |  |  |  |  |
| TSPAN33 | NP_848657.1 | 100 | 97 | 87 | 44 | 99 | 73 | 92 | 34 | 90 | 41 |  |  |

**Table 1. The evolutionary conservation of tetraspanins in different species**

*: 33 human tetraspanins are blasted with “blastp” program from NCBI. C: percentage of query sequences covered. I: percentage of identity. No data is generated when blasted with Arabidopsis.
