# Supplementary material for: Expression and Function of Tetraspanins and Their Interacting Partners in B Cells
Source: Front Immunol. 2018 Jul 18;9:1606. doi: 10.3389/fimmu.2018.01606 (PMC6058033; doi:10.3389/fimmu.2018.01606)
Supplement: Supplementary file 4 [file table_4.DOCX]

**Table 4. The categories of proteins associating with tetraspanins**

| Categories | Partners of tetraspanins |
| --- | --- |
| Tetraspanins | CD151, CD37, CD53, CD63, CD81, CD82, CD9, ROM1, TSPAN2, TSPAN3, TSPAN12 |
| Integrin | ITGA2, ITGA4, ITGA5, ITGA6, ITGB1, ITGB3, ITGB4, ITGA3, ITGA7 |
| G-protein coupled receptor | ADGRE5, ADGRG5, AGTR1, C3AR1, FZD1, FZD10,FZD3, FZD4, GLP1R, GPR141, LPAR1, LPAR6, P2RY12 |
| Transcription regulator | CREB3, HNF4A, HNRNPD, NFKB1, NFKBIB, PDX1, RBL1, REST, TFCP2, THAP11 |
| Transporter | AP3M1, ARF6, ATP13A2, ATP1A1, ATP2A3, ATP2B3, EPN1,GABARAP, REEP5, SLC22A16, SLC44A1, SLC7A1, SNX13, SNX14  SNX17, STX6, TM9SF4, TMED10, TMEM30B, TUSC3 |
| Peptidase/phosphatase | ADAM10, ECEL1, EPHX1, RNF130, TMPRSS11B, PTPN6 |
| Kinase | EGFR, IPPK, LYN, MET, NRP2, PI4KA, PIK3CA, PIK3CD, PIK3CG, PIK3R2, PIK3R3, PRKCA, PRKCB, SYK |
| Ion channel | ASIC4, CACNA1A, CLCC1, HTR3A, LRRC8A, PIEZO1 |
| Enzyme | ADCY5, AGPAT3, CDS1, CYB5R3, DAGLB, DHRS7, DPY30, GGT1, HSD17B13, HSDL1, IFIH1, KARS, LCLAT1, LPCAT3, PHGDH, PIGO  PNLDC1, PNPLA6, RAC2, RDH14, RNF13, RNF149, SOAT1, SPTLC2, TMEM62, UGCG, VNN2, ZDHHC6 |
| Other transmembrane or plasma membrane proteins | LRP5,PRPH2, TNFRSF10B, TNFRSF17, CD19, CD2, CD36, CR2, APP, CD1D, CD44, CLDN1, CLDN11, CLDN7, CLEC5A, EPCAM, EZR, LGALS9, GYPB, GP1BB, HAVCR2, IGLL1, IGSF8, KPTN, LRRTM1, MSN, PTGFRN, REEP6 |
| Other nucleus proteins | CIB1, CDC6, CCDC155, TMEM185A |
| Other cytoplasm proteins | ACTA1, ARL6IP5, BNIP2, BCL2L13, BSCL2, CCDC115, ELAVL1, ESYT1, FITM2, GHDC, GOLGA7, ITSN1, KLHL2, MAVS, MTCH1, PLEKHA7, SERPINA12, SNX25, TMEM173, TMEM87A, UPK3A |
| Other extracellular  space proteins | TIMP1, NDP, BBS1, FAM210B, LGALS3, LGALS8, NAT14, PDZD11, POMC |
| Others | ALG11, DIRC2, DPY19L1, EVA1C, FAM189B, FAM241A, GRAMD1C, LMF2, LYPD4, MAP1LC3B2, PRAF2, RETREG3, SNX31, SYPL2, TVP23C, TMEM223, TMEM39A, TYW1 |
